# Cytoreductive surgery and hyperthermic intraperitoneal chemotherapy for peritoneal malignant tumors in children: Initial experience in a single institution

**DOI:** 10.3389/fsurg.2022.1078039

**Published:** 2023-01-11

**Authors:** Zhiyun Zhu, Xiaofeng Chang, Jiarong Wang, Shen Yang, Hong Qin, Wei Yang, Haiyan Cheng, Deguang Meng, Huanmin Wang

**Affiliations:** ^1^ Department of Surgical Oncology, Beijing Children's Hospital, Capital Medical University, National Center for Children's Health, Beijing, China; ^2^ Department of Surgical Oncology, Baoding Branch of Beijing Children's Hospital, Baoding Children's Hospital, Baoding, China

**Keywords:** cytoreductive surgery, hyperthermic intraperitoneal chemotherapy, child, malignant tumor, safety

## Abstract

**Background:**

Peritoneal malignant tumors in children are rare but commonly associated with disease progression and poor outcome. The successful treatment experience of cytoreductive surgery (CRS) and hyperthermic intraperitoneal chemotherapy (HIPEC) in adult peritoneal carcinoma has been applied to pediatric peritoneal malignancy in recent years. However, patients with desmoplastic small round cell tumor (DSRCT) accounted for the majority of patients treated with CRS and HIPEC in previous studies. The role of CRS and HIPEC remains controversial due to the rarity of the disease and the limited sample size of studies. Additionally, the cases using CRS and HIPEC except DSRCT were mainly small case reports with unclear outcomes. We present our experience in the treatment of pediatric peritoneal malignancies using CRS and HIPEC, with more emphasis on the safety, feasibility, and short-term outcome.

**Methods:**

A retrospective query from December 2019 to February 2022 identified 19 children with peritoneal malignancies who underwent CRS and HIPEC in our institution. Clinical characteristics, therapies, and outcomes were summarized and analyzed.

**Results:**

The median age of the patients was 6.4 years (range, 0.7–13.9 years). The histologic types included rhabdomyosarcoma (7), Wilms tumor (2), clear cell sarcoma of the kidney (2), undifferentiated sarcoma (2), immature teratoma (1), peritoneal serous carcinoma (1), malignant rhabdoid of the kidney (1), malignant germ cell tumor (1), neuroblastoma (1), and epithelioid inflammatory myofibroblast sarcoma (1). Seven patients underwent initial operation, and 12 patients received reoperation for tumor recurrence. The median peritoneal carcinomatosis index was 5 (range, 2–21). There were no perioperative deaths or life-threatening complications of CRS and HIPEC. Two patients had grade 3 complications of wound infection and wound dehiscence. With a median follow-up time of 14 months (range, 1.5–31 months), 14 patients were alive, and 5 died of tumor recurrence. Of the 14 patients who were alive, 2 relapsed after CRS and HIPEC and then received radiotherapy and molecular-targeted therapy or chemotherapy.

**Conclusions:**

CRS and HIPEC are safe and feasible in children, without increasing serious complications in the peri- and postoperative periods. The complication is acceptable. The short-term outcome shows possible effectiveness in pediatric peritoneal malignant tumors. The long-term effectiveness needs to be verified by additional cases and long-term follow-ups.

## Introduction

Peritoneal malignant tumors are rare in children and commonly present with peritoneal sarcomatosis (PSC). Extensive disseminated peritoneal malignancies are usually managed aggressively as high-risk disease with poor outcome. The successful experience of cytoreductive surgery (CRS) and hyperthermic intraperitoneal chemotherapy (HIPEC) in adult peritoneal carcinoma (PC) has been applied to treat pediatric peritoneal malignancy in recent years and demonstrated possible survival benefits (1–3). Due to the rarity of pediatric peritoneal malignancy and the limited sample size of previous studies, CRS and HIPEC were mainly reported as small case series and few case reports. The role of CRS and HIPEC in children remains controversial (3–5). Hayes-Jordan et al. began the first pediatric HIPEC program and reported the largest numbers of pediatric CRS and HIPEC procedures, which demonstrated that patients with DSRCT had longer survival than those with other tumors in children (1, 4). On the other hand, a retrospective French national study analyzed 22 pediatric patients with peritoneal tumors treated by CRS and HIPEC over 14 years and did not find a significant independent effect of HIPEC for DSRCT. However, patients with mesothelioma obtained more benefits by CRS and HIPEC (3). CRS and HIPEC also demonstrate some benefits in other histologic types (2, 6–9), but only in small sample size studies and case reports showing uncertain effects. Moreover, the safety and feasibility data of CRS and HIPEC in children have rarely been reported. Starting in 2019, we have been performing CRS and HIPEC for the management of pediatric peritoneal malignancies in our institution and paying more attention to the safety and feasibility in children and the short-term outcome in this study.

## Methods

A retrospective review was performed for patients with peritoneal malignant tumors treated by CRS and HIPEC in our institution from December 2019 to February 2022. Data were collected from the medical files.

The inclusion criteria were as follows: (1) age < 18 years old, (2) good performance status, (3) normal liver and renal functions, (4) diseases limited to the abdominal cavity, and (5) response to initial chemotherapy. Ethical approval was obtained from the appropriate institutional ethics boards.

CRS and HIPEC were a part of the multimodal therapy for these patients. All patients had been treated with neoadjuvant chemotherapy and achieved a partial response before surgery. For most patients, neoadjuvant chemotherapy used the Children's Oncology Group (COG) protocol according to pathological types, stages, and risk stratification. For patients who relapsed more than once, individualized chemotherapy was used.

After the whole abdominal exploration during the operation, the burden of peritoneal disease was evaluated by the intraoperative peritoneal cancer index (PCI) of Harmon and Sugarbaker (10). CRS was performed to remove the visible tumors. When the patient underwent surgery with complete cytoreduction (CC0/CC1) (10), HIPEC with the open technique at 40.5–41.5°C for 60 min was performed. The chemotherapy regimens of HIPEC were as follows: (1) doxorubicin + cisplatin, (2) doxorubicin + ifosfamide, and (3) cisplatin. The detailed doses were doxorubicin 15 mg/m^2^, cisplatin 50 mg/m^2^, and ifosfamide 1 g/m^2^. The inflow and outflow cannulas were placed below the diaphragm and in the pelvic cavity. Vital signs were monitored during the surgery.

The postoperative cardiac, liver, renal function, and gastrointestinal recovery were recorded. Complications were graded by the National Cancer Institute's Common Terminology Criteria for Adverse Events (NCI-CTCAE) v4.0 (https://ctep.cancer.gov/protocolDevelopment/electronic_applications/ctc.htm#ctc_50). The postoperative treatment and survival status of all children were followed up. Disease-free survival (DFS) was the time from surgery (HIPEC + CRS) to tumor recurrence or death or the last follow-up.

## Results

### Clinical characteristics

Nineteen patients (11 males and 8 females) with peritoneal malignancy were enrolled in this study. The median age was 6.4 years (range, 0.7–13.9 years). Seven patients underwent an initial operation (2 patients with preoperative rupture), and 12 patients underwent reoperation (2 patients with primary preoperative rupture) for tumor relapse. The histologic types included rhabdomyosarcoma (7), Wilms tumor (2), clear cell sarcoma of the kidney (2), undifferentiated sarcoma (2), immature teratoma (1), peritoneal serous carcinoma (1), malignant rhabdoid of kidney (1), malignant germ cell tumor (1), neuroblastoma (1), and epithelioid inflammatory myofibroblast sarcoma (1). The characteristics of all the patients are shown in Table 1.

**Table 1:** Patient characteristics, treatments, and short-time outcomes.

Patient #	Gender	Age (years)	recurrent times	Diagnosis	Preoperative rupture	Procedure	PCI	CCR	HIPEC regime	Operating time (h)	Postoperative management	Last follow -up (m)	Status	Recurrence	Time to recurrence (m)	DFS
1	Male	1.8	2	Nephroblastoma	No	Partial hepatectomy, partial peritonectomy	4	0	DOX, IFO	6.7	Chemotherapy	31	NED	No	—	31
2	Female	7.2	2	Undifferentiated sarcoma	No	Partial hepatectomy, partial peritonectomy	5	0	DOX, IFO	5.7	Chemotherapy	10	DOD	Diaphragm	2	2
3	Male	1.7	0	Rhabdomyosarcoma	No	Partial peritonectomy, pelvic lymphadenectomy	2	0	DOX, DDP	7.2	Chemotherapy, radiotherapy	23.5	NED	No	—	23.5
4	Male	1.7	2	Malignant rhabdoid of kidney	Yes	Partial peritonectomy	2	1	DOX, DDP	5.8	Chemotherapy	3.5	DOD	Retroperitoneum	2.5	2.5
5	Male	0.7	1	Immature teratoma	No	Partial peritonectomy, partial mesocolic excision	9	0	DDP	6.5	Chemotherapy	20	NED	No	—	20
6	Male	5.3	1	Neuroblastoma	No	Partial peritonectomy	7	1	DDP	7.8	Chemotherapy	8	DOD	Retroperitoneum	7	7
7	Female	3.8	0	Peritoneal serous carcinoma	No	Extensive peritonectomy, omentectomy	21	1	DOX, DDP	9.2	Chemotherapy, radiotherapy, targeted therapy	19.5	NED	Retroperitoneum	7.5	7.5
8	Female	5.6	0	Rhabdomyosarcoma	No	Partial peritonectomy, omentectomy	3	0	DOX, IFO	7	Chemotherapy, radiotherapy	19	NED	No	—	19
9	Male	13.9	1	Rhabdomyosarcoma	No	Partial peritonectomy, omentectomy	6	1	DOX, IFO	6.8	Chemotherapy, radiotherapy	18.3	AWD	Peritoneum	17.5	17.5
10	Female	9.2	0	Rhabdomyosarcoma	Yes	Partial peritonectomy, partial omentectomy	2	0	DOX, DDP	8.2	Chemotherapy, radiotherapy	15.5	NED	No	—	15.5
11	Female	12.9	0	Malignant germ cell tumor	Yes	Partial peritonectomy, omentectomy	2	0	DDP	6	Chemotherapy	14	NED	No	—	14
12	Male	2.8	1	Rhabdomyosarcoma	No	Partial peritonectomy	3	0	DOX, IFO	7	Chemotherapy, radiotherapy	13.2	NED	No	—	13.2
13	Male	7.7	4	Undifferentiated sarcoma	No	Partial peritonectomy, omentectomy, partial mesenterectomy	11	0	DOX, IFO	7	Chemotherapy, radiotherapy	12	DOD	Peritoneum	10	10
14	Female	8.2	0	Rhabdomyosarcoma	No	Extensive peritonectomy, omentectomy	12	0	DOX, IFO	6	Chemotherapy, radiotherapy	12.5	NED	No	—	12.5
15	Female	9.8	0	Rhabdomyosarcoma	No	Partial peritonectomy	9	1	DOX, IFO	6	Chemotherapy, radiotherapy	12	NED	No		12
16	Female	6.4	1	Clear cell sarcoma of the kidney	Yes	Partial peritonectomy, partial gastrectomy	7	0	DOX, IFO	6.3	Chemotherapy, radiotherapy	12	NED	No	—	12
17	Female	5.4	2	Nephroblastoma	Unknown	Partial peritonectomy, partial sigmoidectomy	8	1	DOX, DDP	7.3	—	1.5	DOD	Peritoneum	1	1
18	Female	3	1	Clear cell sarcoma of the kidney	No	Partial peritonectomy	6	0	DOX, IFO	4.5	Chemotherapy, radiotherapy	9.3	NED	No	—	9.3
19	Female	5.3	1	Epithelioid inflammatory myofibroblast sarcoma	No	Partial peritonectomy, partial omentectomy	2	0	DOX, IFO	5	Chemotherapy, radiotherapy, targeted therapy	5	NED	No	—	5

AWD, alive with disease; NED, no evidence of disease; DOD, dead of disease; DOX, doxorubicin; IFO, ifosfamide; DDP, cisplatin; PCI, peritoneal cancer index; CCR, completeness of cytoreduction; HIPEC, hyperthermic intraperitoneal chemotherapy.

### CRS + HIPEC and complications

The median PCI was 5 (range, 2–21), and the CC score was 0–1. The median operation time was 6.7 h (range, 4.5–10.5 h). One patient underwent partial sigmoidectomy and colostomy, 1 patient underwent partial gastrectomy, 2 patients underwent partial hepatectomy, and 1 patient underwent partial cystectomy. The other 14 patients had no organ resection.

There were no perioperative deaths or serious adverse events related to CRS and HIPEC in our study. Increased alanine transaminase (ALT) was found in seven cases and returned to the normal limit within 11 days. In two cases, serum creatine kinase isoenzyme (CK-MB) was two times higher than the normal value and returned to less than two times within 7 days after the operation, and no cardiac dysfunction was observed. No renal dysfunction occurred. All patients were routinely placed with gastric tubes after CRS and HIPEC. The gastric tube was removed 6 days after surgery in the patient with colostomy, 7 days after surgery in the patient with partial gastrectomy, and 2–3 days after surgery in the other 17 patients. All patients received oral feeding well after the operation. No bowel obstruction occurred.

Among 19 patients, two patients had grade 3 complications. One patient had a wound infection and needed intravenous antibiotics. The other patient had wound dehiscence that required suturing under general anesthesia 2 weeks after CRS and HIPEC. There were no other severe HIPEC-associated toxicities or complications observed.

### Postoperative treatment and follow-ups

All 19 patients received chemotherapy (*n* = 18) and radiotherapy (*n* = 12) according to the different diagnoses. The chemotherapy used the COG protocol or individualized adjuvant chemotherapy. Two patients received molecular-targeted drugs. All patients were followed up. The median follow-up time was 12.5 months (range, 1.5–31 months). Fourteen patients were alive, and five died of tumor recurrence. Of the 14 patients who were alive, 2 relapsed after CRS and HIPEC and then received radiotherapy and molecular-targeted drugs or chemotherapy. The other 12 patients had no sign of recurrence. Of the seven patients with recurrence, five patients discontinued treatments and died, one patient received radiotherapy and molecular-targeted drugs and was alive without disease, and one patient was undergoing chemotherapy and was alive with disease. The median DFS was 12 months (range, 1–31 months). Table 1 shows the treatment and follow-ups of 19 patients treated with CRS and HIPEC.

## Discussion

This report described the early experience of CRS and HIPEC in the treatment of pediatric peritoneal malignant tumors in our institute, one of the largest childcare centers in China. This finding supports that this aggressive surgery is safe and feasible in children, with a limited patient sample. Although two patients suffered from grade 3 complications, it was acceptable. There was no significant organ dysfunction after surgery. CRS and HIPEC may play a role in the local control of pediatric rare malignant peritoneal tumors in the short term.

Peritoneal malignant tumors are uncommon in children with unknown incidence. Pediatric peritoneal malignancy usually manifests as PSC (11, 12). In comparison, PCs are more common in adults. Systemic chemotherapy has little effect on peritoneal malignancy because of the limitation of peritoneal penetration. The recurrence rate of peritoneal disease is higher than that of other anatomic sites and lacks standard treatment. Peritoneal malignancy in children is usually treated as a high-risk disease with multimodal therapy, including chemotherapy, surgery, and radiotherapy, but still has a poor long-term prognosis (1, 4).

Peritoneal malignancy is most commonly metastatic in children, usually after the neoplasm breaks through the organ capsule, through hematogenous dissemination of primary tumors, tumor spillage from incomplete surgical resection, or local spread of malignant peritoneal tumors. Effective treatment for free tumor cells and microscopic disease in the peritoneal cavity after surgery is a strategy to prevent peritoneal dissemination. In recent years, CRS and HIPEC, as successful local control approaches in adult PC, have been applied to pediatric peritoneal malignancies. The majority of reports are in children with DSRCT (1, 3, 4, 13, 14, 16).

For patients with pediatric peritoneal mesothelioma, studies in the literature have reported a positive effect of HIPEC. Scalabre et al. demonstrated that pediatric patients with peritoneal mesothelioma treated by CRS and HIPEC had a significantly better overall survival (OS) (*p* = 0.015) and DFS (*p* = 0.028) in mesothelioma (*n* = 7) than other histological types (3). A retrospective study investigated a prospective database including patients with malignant peritoneal mesothelioma who underwent CRS and HIPEC from 1994 through 2014 in the United States. A total of 71% (5/7) of patients were reported to be alive more than 5 years after treatment; however, the historic median survival was 11 months in young patients (15). However, tumors with other histologic types treated with CRS and HIPEC were small case series, and the effect is uncertain, including rhabdomyosarcoma, angiosarcoma, and colon cancer (2, 4, 6–9, 16).

In our study, more than half of the cases (10/19) presented with peritoneal sarcomatosis, and the other nine cases presented with various histologic types, including a case of peritoneal adenocarcinoma. There were 12 patients with postoperative recurrence before CRS and HIPEC, most of whom were treated with multimodal therapy, including chemotherapy and radiotherapy, and even molecular-targeted drugs in some patients. Two of them relapsed four times. Therefore, we performed this study to explore the treatment for these troublesome diseases. The preliminary results showed that 6 of the 12 patients (50%) had no recurrence after CRS and HIPEC, and the median DFS was 8.7 months. The other six patients with initial surgery had no evidence of disease except 1onepatient with diffuse peritoneal adenocarcinoma, who relapsed 7.5 months after CRS and HIPEC and was alive without disease after receiving molecular-targeted drug and radiotherapy. Although five patients died from relapse after CRS and HIPEC, they belonged to those who relapsed at least one time and were difficult to treat. The short-term outcome showed possible local control and possible survival benefits of CRS and HIPEC in our study. However, the follow-up period was short, and longer follow-up is needed to determine the long-term effect.

CRS and HIPEC are aggressive operations, and the operative morbidity reported in adults is approximately 40%. However, the morbidity in children is lower. The common complications reported in the literature in children were nephrotoxicity, neurogenic bladder, urinary tract infections, wound infection, abscess and enterocutaneous fistula, and partial small bowel obstruction (1, 3, 17). In our study, no small bowel obstruction or nephrotoxicity was observed. Two patients had grade 3 complications of wound infection and wound dehiscence after CRS and HIPEC.

Limitations of this study include various pathological types, relatively few cases of each type, and a short follow-up time. This is mainly due to the rare incidence of peritoneal tumors in children and the short period in which we have been performing this approach. For future work, it is necessary to cooperate with multiple centers, expand the sample size, conduct prospective studies, extend the follow-up time, and obtain long-term results.

In conclusion, CRS and HIPEC procedures are safe and feasible in children, without increasing serious complications in the peri- and postoperative periods. The adverse event is acceptable. The short-term outcome showed possible effectiveness in pediatric peritoneal malignant tumors. The long-term effectiveness needs to be verified by additional cases and long-term follow-ups.

## Data Availability

The original contributions presented in the study are included in the article/Supplementary Material, further inquiries can be directed to the corresponding authors.
